# Energy-efficient path planning for Robotic Bulkhead Inspection using Residual-Enhanced UKF and Hierarchical MPC

**DOI:** 10.1371/journal.pone.0342222

**Published:** 2026-03-19

**Authors:** Jiexin Wang, Lei Li, Runlin Gao, Liu Yang

**Affiliations:** College of Mechanical Engineering, Jiangsu University of Science and Technology, Zhenjiang, Jiangsu, China; Aalto University, FINLAND

## Abstract

Ultrasonic thickness inspection of ship bulkheads poses significant challenges due to confined spaces, dynamic obstacles, and highly variable environments. This paper presents a novel autonomous robotic arm control framework tailored for such conditions, combining enhanced Unscented Kalman Filter (UKF) with a hierarchical Model Predictive Control (MPC) strategy. We introduce a residual-driven adaptive noise covariance UKF (RD-ANC) integrated with a Huber penalty function (HP-UKF), significantly improving robustness against sensor noise and outliers during real-time mapping and estimation. A Three-Layer Energy-Efficient MPC (TLE-MPC) is designed, comprising: a global planner using Differential Dynamic Programming (DDP) for energy budgeting and coarse path generation; a coordination layer using Sequential Quadratic Programming (SQP) for obstacle avoidance and adaptive energy trade-offs; and an execution layer leveraging Explicit MPC (eMPC) for sub-5 ms control law computation. Simulation results show the framework achieves real-time obstacle avoidance, stable path tracking, and up to 15% energy reduction during inspection tasks in semi-structured and unpredictable ship environments. This research offers a robust and scalable method for autonomous robotic inspection and lays the foundation for future multi-arm cooperation and long-duration energy-aware deployments.

## 1. Introduction

Robotic arms have seen increasing adoption in ship assembly, maintenance, and inspection due to their ability to perform high-precision tasks in challenging environments [1]. One such critical task is ultrasonic thickness measurement of ship bulkheads, which ensures structural integrity [2]. However, this task remains difficult to automate because it must be performed in semi-structured, cluttered, and dynamic compartments, such as ballast tanks and rainwater storage units [3]. These areas often contain unpredictable obstacles like temporary scaffolding, aged coatings, or emergency repair pipelines, making reliance on pre-built maps impractical.

Human-conducted inspections in such environments are labor-intensive, expose workers to safety risks at height, and are subject to human error over repetitive tasks. While industrial robots offer automation potential, most rely on static maps and offline planning, struggling to adapt to dynamic changes in the environment. The difficulty in industrial robots primarily stems from the semi-structured and unknown nature of the operational environment [4]. A semi-structured environment contains both fixed structural elements and randomly changing, unstructured components. The structured elements include bulkheads, ribs, and pipes, whose geometric outlines follow classification society and International Maritime Organization (IMO) standards. These elements adhere to engineering guidelines, allowing their layout to be predicted using Computer-aided design/ Strategic Information Systems Planning model (CAD/SISP) models supported by prior knowledge.

Conversely, the unstructured components involve temporarily installed bypass pipelines for emergency repairs or equipment upgrades, aging protective coatings exhibiting blistering or flaking, scaffolding erected during maintenance, and gas cylinders stacked temporarily. Such scenarios make reliance on pre-built maps infeasible, necessitating real-time perception capabilities. Consequently, different ships—or even the same ship at various stages of maintenance—exhibit significantly different internal environments [5]. Traditional robots relying on static maps cannot promptly respond to such dynamic changes and thus struggle with path planning in unknown environments [6]. At present, most industrial robots depend heavily on predefined maps for trajectory planning; encountering unknown obstacles forces them to halt and replan their paths, significantly reducing operational efficiency [7]. As a result, manual labor remains a substantial component in ship inspection tasks.

Thickness measurements on bulkheads often require working at height, which not only poses considerable safety risks but also heavily depends on operator experience. Additionally, human inspection processes over prolonged and repetitive tasks exhibit a notably higher error rate compared to automated systems [8]. Thus, developing a robotic arm path-planning framework capable of real-time perception, robust state estimation, and instantaneous obstacle avoidance has urgent and practical significance in improving the safety and efficiency of ship bulkhead inspections.

In recent years, robotic arms have seen increasing adoption in ship assembly and maintenance, with robotic manipulators capable of autonomous path planning gaining significant popularity [9]. Integration with various inspection tools has further empowered robotic arms to undertake diverse operational tasks. Junfei Li [10] proposed a knowledge-based genetic algorithm designed to generate collision-free paths within complex environments. Xin Cheng et al. [11] developed an enhanced RRT-Connect algorithm featuring adaptive step-size strategies and fixed sampling methods by constructing four randomized trees from start points, endpoints, and fixed points. Similarly, R. T. Chien et al. [12] introduced a topological method utilizing Reachable Manifold Graphs (RMG), leveraging the concepts of state space and rotational mapping to achieve collision-free translational and rotational movements of robotic arms among obstacles. Although various path-planning algorithms have demonstrated strong performance in their respective fields, issues persist, notably high dependence on pre-existing maps and substantial computational loads.

Hubo Chut [13] explored predictive-model-based collaborative robotic arms, introducing a proactive collaborative controller informed by human multimodal information. Jingmou Nie [14] addressed high-precision tracking and dynamic obstacle avoidance for mobile robotic arms with multiple constraints, proposing an innovative method combining Model Predictive Control (MPC) and Prescribed Performance Functions (PPF). Additionally, deep learning methods have shown promising results in learning high-dimensional control tasks, yet the significant consumption of unnecessary samples during training remains problematic. To address this, Mostafa Al-Gabalawy [15] developed a hybrid structured algorithm combining MPC and Deep Reinforcement Learning (DRL), termed MPC-DRL.

MPC, extensively utilized in fields such as chemical engineering and automotive control, has witnessed growing adoption in robotic arms due to advances in computational efficiency and predictive capabilities [16]. Its inherent predictive nature is particularly suitable for path planning in unknown environments. Zhiqiang Zuo [17] proposed a Progressive MPC Scheme (PMPCS), introducing an improved Particle Swarm Optimization (IPSO)-based MPC to effectively resolve planning and tracking problems. Additionally, Zanyu Tang [18] introduced a novel minimal movement scheme, the Minimum Movement Planning and Control (MMPC) method, enabling robotic arms to minimize unnecessary movements efficiently.

Recent advances have further enhanced MPC’s adaptability to uncertainty, including sensitivity-aware and chance-constrained formulations that accommodate model inaccuracies and sensor noise [19,20]. To address localization errors under uncertainty, Kalman filter variants such as the Extended and Unscented Kalman Filters (EKF, UKF) have been widely used for sensor fusion and state estimation in mobile and articulated robots [21,22]. However, these methods often assume fixed noise covariance and are sensitive to outliers, limiting their robustness in highly dynamic environments like ship compartments.

Some researchers have has explored hybrid and layered control architectures that combine global planning with local real-time correction. For instance, hierarchical MPC frameworks have been applied to integrate global route generation with fast, constraint-aware execution [23,24]. Others have introduced learning-enhanced estimation and control pipelines, blending classical methods with neural approximators to improve performance in changing conditions [25].

Despite the successful application of MPC, its strong dependence on model accuracy means that even minor sensor noise or parameter inaccuracies can significantly degrade control performance [26]. Dynamic parameter changes and measurement inaccuracies often lead to computational deviations and practical errors. To address this issue, the Unscented Kalman Filter (UKF) has been introduced to enhance sensor data accuracy and mitigate noise [27,28]. State estimations processed by UKF can substantially improve system precision and robustness. Minhan Li [29] proposed a model-free method based on an adaptive Kalman filter, enabling path tracking of continuum robots using only pressure and end-effector position data.

Researchers have also actively explored various approaches combining Kalman filtering with predictive control, such as adaptive Model Predictive Controllers integrated with Kalman filters [29] and nonlinear MPC methods based on Extended Kalman Filters (EKF) [30]. Wang Q et al. [17] developed an MPC algorithm enhanced with Kalman Filtering to tackle issues arising from environmental disturbances, sensor noise, and time-varying velocity instabilities. Additionally, Jiading Bao et al. [31] constructed a UKF-MPC-based controller to enhance overall system robustness, facilitating low-latency autonomous navigation in robotic swarms.

### 1.1. Research Gaps

Although previous work has proposed various path-planning strategies—such as RRT-based sampling, topological graphs, and deep reinforcement learning—most approaches depend on known or pre-mapped environments, ignore energy consumption during path planning, and lack robustness to real-time sensor noise and environmental uncertainty. Moreover, while MPC has gained popularity for its predictive capabilities, it is sensitive to model inaccuracies and sensor noise. Kalman filtering (e.g., UKF and EKF) has been explored to improve estimation, but often lacks dynamic noise adaptation, leading to degraded performance in rapidly changing inspection settings.

### 1.2. Contributions

To address the challenges of robotic ultrasonic inspection in dynamic, semi-structured ship environments, this study presents a novel autonomous path-planning framework based on UKF and hierarchical MPC. The key contributions include:

1. We propose a residual-driven adaptive noise covariance (RD-ANC) approach that dynamically adjusts both process and observation noise using real-time residuals. Coupled with a Huber penalty function (HP-UKF), this significantly enhances robustness to LiDAR outliers and sensor noise in dynamic inspection scenarios.
2. A hierarchical control architecture is designed with three cooperative layers:

- A global layer using Differential Dynamic Programming (DDP) for task decomposition and energy budgeting
- A coordination layer using Sequential Quadratic Programming (SQP) for real-time obstacle avoidance and adaptive energy trade-offs
- An execution layer using Explicit MPC (eMPC) for sub-5 ms trajectory execution with actuator constraints

This layered design ensures energy efficiency, fast responsiveness, and safe path tracking in unstructured compartments.

1. The framework integrates solid-state LiDAR and encoder signals at 50 Hz, enabling replanning within 100 ms when encountering dynamic obstacles. This supports autonomous operation even in rapidly changing internal ship environments.
2. We validate the full UKF + TLE-MPC system in a simulated ship compartment environment using MATLAB. Results demonstrate smooth, efficient path tracking with up to 15% energy savings and robust performance under motion uncertainty and obstacle disturbances.

Together, these contributions form a unified control system for robotic arms operating in dynamic, map-scarce environments — providing a reproducible foundation for future multi-arm collaboration and long-duration inspection tasks.

The structure of the rest of the paper is as follows: Section II describes the specific framework of the UKF-MPC and provides detailed designs of both the UKF and MPC modules. Section III presents simulation and verification experiments to evaluate the performance of the proposed UKF-MPC algorithm. Finally, Section IV concludes with a summary of the key contributions and findings.

## 2. Methodology

### 2.1. System framework

**Fig 1:**
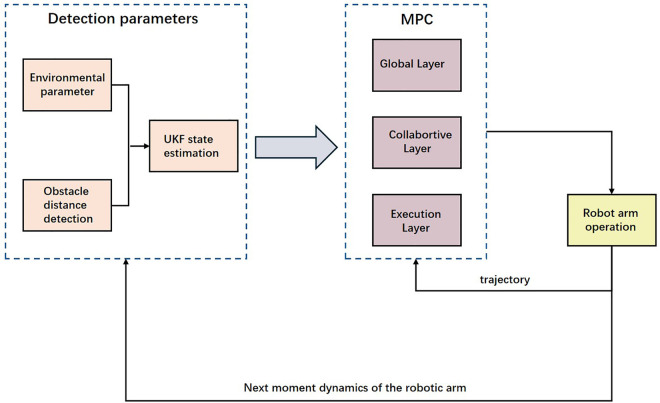
Proposed UKF-MPC based operation of Robotic arm.

This paper focuses on ultrasonic thickness measurement tasks conducted by robotic arms within complex and dynamic ship compartment environments, including ballast tanks, rainwater tanks, oil tanks, and external hull surfaces. Given the highly variable operational scenarios and substantially different working trajectories encountered by the robotic arm, simultaneous capabilities for global path planning and real-time obstacle avoidance are essential to achieve comprehensive autonomous operations. In response to these requirements, this study proposes an integrated UKF-MPC path planning framework, capable of real-time data observation, path prediction, and dynamic planning. The core of the proposed framework is illustrated in Fig 1 below:

1. Environmental Perception: Solid-state LiDAR collects point cloud data at 50 Hz, calibrating initial inspection points. Upon detection of unknown obstacles, the system calculates the minimum real-time distance between the robotic arm’s end-effector and obstacle surfaces.
2. State Estimation: LiDAR point clouds and encoder signals are transmitted via bus communication to the central controller, where a UKF fuses multi-source information at 50 Hz. The introduced RD-ANC and HP-UKF methods effectively suppress dynamic noise and outliers, significantly enhancing estimation accuracy.
3. Hierarchical Energy-efficient MPC (TLE-MPC):The global layer employs Differential Dynamic Programming (DDP) every 1 second to recompute global tasks and energy budgets. The coordination layer updates relaxed obstacle avoidance constraints and adaptive energy-consumption weighting at 5 Hz based on local map data. Finally, the execution layer utilizes explicit MPC (eMPC) at 200 Hz to generate instantaneous optimal trajectories subject to actuator saturation and a 30 mm safety constraint.

Leveraging this multi-timescale collaborative approach, the robotic arm maintains robust end-effector speed within complex compartments, expecting an overall energy consumption reduction of approximately 15%. Moreover, the system is capable of replanning trajectories within 100 ms when unexpected obstructions occur, ensuring rapid task resumption.

### 2.2. Environmental awareness and UKF state estimation

For a general n-degree-of-freedom (DOF) robotic manipulator, its dynamics can be modeled using the Lagrangian method, where the Lagrangian function is defined as:


$$L(q,\dot{q})=K(q,\dot{q})-P(q)$$ (1)

where $q\in {\mathbb{R}}^{n}$ is the joint position vector, $\dot{q}\in {\mathbb{R}}^{n}$ is the joint velocity vector, $K(q,\dot{q})$ represents the total kinetic energy, and P(q) is the total potential energy of the manipulator system. Thus, the Lagrangian dynamic equation of the manipulator is:


$$M\left(q\right)\ddot{q}+C\left(q,\dot{q}\right)\dot{q}+G\left(q\right)=\tau +J^{T}\left(q\right)f_{r}$$ (2)

where

$M\left(q\right)\in R^{n*n}$ is a symmetric, positive-definite inertia matrix;

$C\left(q,\dot{q}\right)\in R^{n}$ represents Coriolis and centrifugal forces;

$G\left(q\right)\in R^{n}$ denotes gravitational forces;

τ is the joint actuation torque vector;

$J^{T}\left(q\right)\in R^{n*n}$ is the transpose of the Jacobian matrix;

$f_{r}$ is the external force applied to the manipulator;

$q,\dot{q},\ddot{q}\in R^{n}$ are the joint position, velocity, and acceleration vectors, respectively.

LiDAR perceives obstacle distance, size, and related information by measuring the time of flight of reflected laser pulses. Traditional mechanical LiDAR systems utilize horizontal rotation to obtain angular measurements. Although they exhibit strong anti-interference capabilities, they are typically expensive and contain heavy moving components. In ship compartment environments, where robotic arms must remain lightweight to achieve higher operational efficiency, such systems are less suitable. In contrast, solid-state LiDAR offers lower cost and a more compact, lightweight structure. With the emergence of new technologies in recent years, its field of view (FOV) has also improved significantly. Therefore, this study adopts solid-state LiDAR as the primary sensor for environmental data acquisition.

LiDAR provides real-time environmental parameters during the robotic arm’s motion, enabling optimal path planning. However, even highly precise LiDAR systems are subject to measurement errors, and the robotic arm itself also introduces uncertainties. To address these issues, this study incorporates the Unscented Kalman Filter (UKF). The primary advantage of the UKF lies in its ability to estimate state predictions and covariances without requiring linearization. Wenling Li [14] proposed a robust Masreliez-Martin UKF, which delivers reliable system state estimations even in the presence of unknown process and measurement noise covariance matrices.

The state and observation equations of this nonlinear system are given by:


x(k+1)=f(x(k),u(k))+w(k) (3)

z(k)=h(x(k),v(k)) (4)

$x\left(k\right)\in R^{n}$ is the system state vector, such as joint angles and velocities of the robotic manipulator;

$u\left(k\right)\in R^{n}$ is the control input;

f(.) represents the manipulator’s dynamic model;

w(k)~N(0,Q(k)), with $Q(k)\in R^{n*n}$ denotes the process noise with covariance matrix Q(k);

$z(k)\in R^{n}$ is the observation vector, e.g., LiDAR data or feature points;

h(.) is the nonlinear observation function of the manipulator;

v(k)~N(0,R(k)), with $R(k)\in R^{n*n}$ denotes the observation noise with covariance matrix R(k)

By applying the Unscented Transformation (UT), the sigma sampling points and their associated weights are obtained as:


$$x^{\left(i\right)}(k/k)=\left\{\begin{array}{c} @c@\hat{x}(k/k)\\ \hat{x}(k/k)+\sqrt{\left(n+\lambda )P(k/k\right)}\\ \hat{x}(k/k)-\sqrt{\left(n+\lambda )P(k/k\right)} \end{array}\right\}$$ (5)

One-step prediction for 2n + 1 sigma point sets:


$$x^{\left(i\right)}(k+1/k)=f[k,x^{i}(k/k)]$$ (6)

One-step prediction of system state quantity:


$$\hat{x}(k+1/k)=\sum_{i=0}^{2n}w^{i}x^{i}(k+1/k)$$ (7)

$$p(k+1/k)\sum_{i=0}^{2n}w^{i}[\hat{x}(k+1/k)-x^{i}(k+1/k)\mathrm{\backslash leftright}[\hat{x}(1+1/k)-x^{i}(k+1/k{)}^{T}]+Q$$ (8)

Use UT again to generate a new set of sigma points:


$${\hat{x}}^{\left(i\right)}(k+1/k)=\left\{\begin{array}{c} @c@\hat{x}(k+1/k)\\ \hat{x}(k+1/k)+\sqrt{\left(n+\lambda )P(k+1/k\right)}\\ \hat{x}(k+1/k)-\sqrt{\left(n+\lambda )P(k+1/k\right)} \end{array}\right\}$$ (9)

The new sigma point set is substituted into the observation equation, and the newly predicted observed quantity is:


$$z^{i}(k+1/k)=h[x^{i}(k+1/k)]$$ (10)

Calculate the new mean and covariance of weighted observation:


$$\begin{array}{c} \bar{z}(k+1/k)=\sum {\mathrm{\backslash nolimits}}_{i=0}^{2n}w^{i}z^{i}(k+1/k) \end{array}$$ (11)

$$\begin{array}{c} P_{z_{k}z_{k}}=\sum {\mathrm{\backslash nolimits}}_{i=0}^{2n}w^{i}[z^{i}(k+1/k)]-[\bar{z}(k+1/k)][z^{i}(k+1/k)]-[\bar{z}(k+1/k){]}^{T}+R \end{array}$$ (12)

$$P_{x_{k}z_{k}}=\sum {\mathrm{\backslash nolimits}}_{i=0}^{2n}w^{i}[x^{i}(k+1/k)]-[\bar{x}(k+1/k)][z^{i}(k+1/k)]-{\left[\bar{z}(k+1/k)\right]}^{T}\mathrm{\backslash ]}$$ (13)

Kalman gain:


$$k(k+1)=P_{x_{k}z_{k}}{P_{z_{k}z_{k}}}^{-1}$$ (14)

System state update and covariance update:


$$\hat{x}(k+1/k+1)=\hat{x}(k+1/k)+k(k+1)[z(k+1)-\hat{z}(k+1/k)]$$ (15)

$$P(k+1/k+1)=P(k+1/k)-k(k+1)P_{z_{k}z_{k}}k^{T}(k+1)$$ (16)

In this study, the motion speed and load of the robotic arm are changing in real time. We hope to adjust quickly when the robotic arm moves at high speed or encounters sudden disturbances. Therefore, a dynamic adjustment factor is introduced and a penalty function is added to enhance the observation update, so as to dynamically adjust the model according to the state of the robotic arm and enhance the robustness.

Residual-driven adaptive noise covariance (RD-ANC) is introduced to dynamically adjust the noise covariance matrix Q and observation noise covariance matrix R by using the prediction residual of UKF:


$$\epsilon_{k}=z_{k}-h\left({\hat{x}}_{k|k-1}\right)$$ (17)

$$Q_{k}=Q_{\text{0}}\cdot (1+\alpha \epsilon_{k}^{\text{2}})$$ (18)

$$R_{k}=R_{\text{0}}\cdot (1+\beta ∥\epsilon_{k}{∥}^{\text{2}})$$ (19)

$\epsilon_{k}$ is the observation residual;

$z_{k}$ is the actual observation value;

h() is a nonlinear observation function;

${\hat{x}}_{k|k-1}$ is based on the observed values of the state at time k−1.

α, β is the adjustment coefficient, which can make the noise covariance larger as the residual is larger, enhancing the robustness to abnormal observations. For scenarios such as ship cabins where noise has a significant impact, the robotic arm can automatically reduce trust in unreliable observations and balance the weights of the model and observations. In this study, we selected α=0.1 and β=0.05 empirically, based on extensive simulation testing. The chosen values provided a good trade-off between filter responsiveness and numerical stability. Higher values of α and β can lead to overestimation of uncertainty, causing sluggish response, while values that are too low reduce the robustness against outliers and model drift. We also observed that these coefficients influence the filter’s convergence speed and tracking accuracy. To ensure stable operation, we bounded the maximum covariance updates and monitored the normalized innovation squared (NIS) as a stability indicator throughout the process. These adaptive mechanisms significantly improved filter robustness in highly dynamic environments typical of ship compartment inspections.

By incorporating Huber penalty based function enhanced observation update (HP-UKF), LiDAR can introduce outliers during observation due to various disturbances, resulting in feature recognition errors. In the original algorithm, a robust kernel function is added to handle outliers:


$$\text{Cost}\left(z_{k}\right)=\sum_{i}\rho \left(\frac{∥z_{k}^{\left(i\right)}-h\left({\hat{x}}_{k}\right)∥}{\sigma }\right)$$ (20)

ρ() chooses Huber kernel function to suppress the influence of large residuals;

σ represents the noise standard deviation, which is estimated through historical data. By reducing the mismatch caused by dynamic obstacles through a penalty function, the accuracy of target point recognition can be improved。

### 2.3. Design and Analysis of TLE-MPC

In different working environments, the paths required for robotic arm operations vary. We expect the robotic arm to continue performing the same tasks even when changing its working environment, which necessitates its ability to autonomously plan paths in unfamiliar environments. MPC endows the robotic arm with the capability of multi-step motion prediction. Combined with sensor feedback, it predicts the outcomes of the robotic arm’s next motion, and subsequently determines its subsequent movement based on environmental parameters.

1. Prediction model

**Fig 2:**
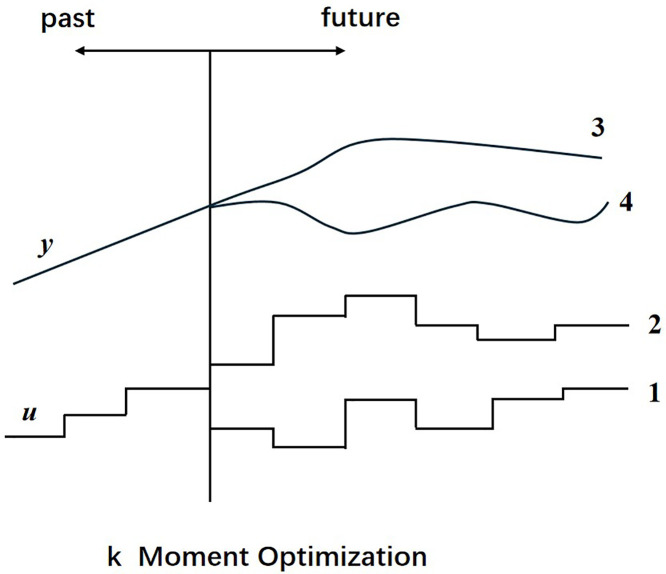
MPC Prediction Model.

Based on the established kinematic model, we predict the system’s performance over a certain future time period to optimize control as shown in Fig 2. This model can reveal the system’s future dynamic behavior and determine the control strategy. By utilizing the predictive model, we can accurately predict the system’s future actions. On this basis, we can further evaluate whether these actions meet the constraints set by the system.

To enable reliable prediction and control, the following boundary conditions and constraints are incorporated into the MPC formulation:

- **Initial state condition:** The prediction starts from the current measured state $x_{\text{0}}=x(t)$, including joint positions and velocities.
- **State constraints:** Joint position, velocity, and acceleration are bounded by robot-specific physical limits, i.e., $x_{min}\leq x_{k}\leq x_{max}$.
- **Control input constraints:** The actuation torques $u_{k}$ are bounded due to actuator saturation, $u_{min}\leq u_{k}\leq u_{max}$.
- **Obstacle avoidance constraint:** A minimum safe distance $d_{safe}=30\;mm$ is maintained between the end-effector and detected obstacles during the prediction horizon.
- **Energy constraint (in coordination layer):** The instantaneous energy consumption is constrained within an adaptive budget $E_{inst}\leq E_{budget}(t)$, dynamically allocated by the global layer.

These constraints ensure that predicted trajectories remain physically feasible, safe, and energy-efficient throughout the motion planning process.

2. Rolling optimization

**Fig 3:**
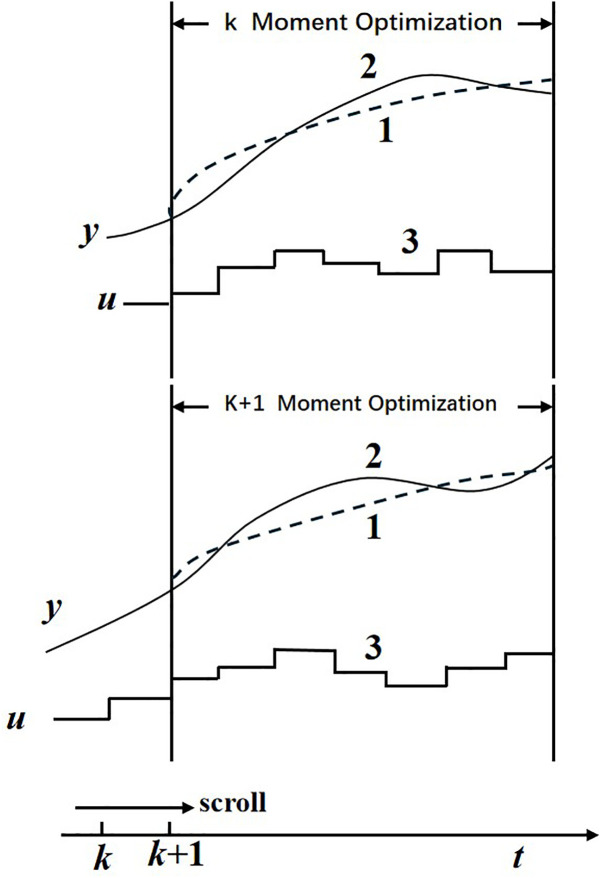
MPC Rolling Optimization Model.

After calculating the optimal control variable, apply one of the control variables to the system. At the next sampling moment, the optimized time domain will roll forward as shown in Fig 3. The optimization process of predictive control is repeated within a limited time range, which is the most significant difference from traditional optimal control methods. Therefore, for systems with frequent changes in dynamic performance and no need to pursue optimal performance on a global scale, rolling optimization methods are most suitable.

3. Feedback correction

**Fig 4:**
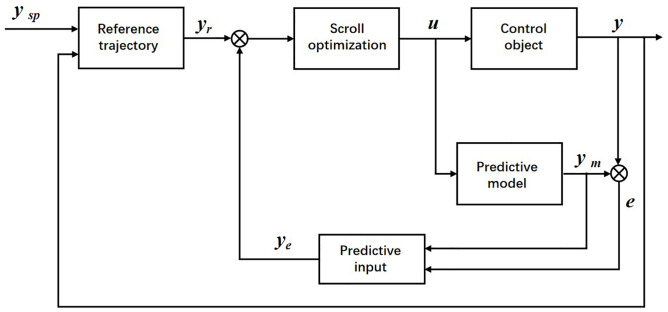
MPC Feedback Correction Model.

MPC, as a feedback control algorithm, can effectively avoid the influence of external disturbances on the control process. However, when the model has matching errors and other issues, it may not achieve the expected optimization effect. Therefore, the model predictive control adopts closed-loop control to reduce errors and improve accuracy as shown in Fig 4.

**Fig 5:**
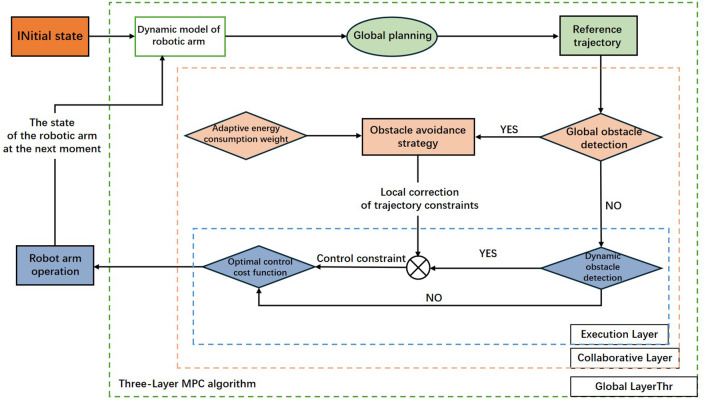
Proposed layered energy-saving MPC for robotic arm operation.

The main drawbacks of traditional MPC control are that linearization and path linearization can lead to prediction errors, and the requirements for the path are also relatively strict. Sven Adrianus Nicolaas Nouwens et al. proposed an approximate ca MPC scheme, which reduces computation time by balancing closed-loop performance and computation time on the basis of the original constrained adaptive MPC (ca MPC); Rabab Benotsmane [16] combines PID and MPC to develop the optimal control strategy for industrial robots, in order to minimize energy consumption to the greatest extent possible. In this study, to address the path planning and trajectory tracking control problems of robotic arms under external disturbances and uncertain parameters, and to achieve better energy consumption and shorter computation time while autonomously planning paths, we propose a layered energy-saving MPC (TLE-MPC) as shown in Fig 5.

#### 2.3.1. System layered architecture.

In this study, the hierarchical architecture of MPC is the core content of the entire work, playing a decisive role in the overall path planning and obstacle avoidance decision-making. The computational efficiency and robustness of the algorithm are the main factors affecting the success rate of robotic arm path planning and directly related to the response time during the obstacle avoidance process. Hierarchical MPC decomposes tasks that require a large amount of computing resources, and each layer architecture analyzes and solves its own optimization problem, significantly improving control stability and accuracy.

(1)The global layer mainly solves the two problems of “where to go” and “when to arrive”. It integrates real-time voxel maps based on CAD/ISP priors and runs differential dynamic programming (DDP) with a period of 1 second to generate a connected, collision free, and approximately shortest coarse-grained end trajectory. At the same time, based on the partition of the cabin and the remaining power of the robotic arm, the allowable energy consumption can be allocated by segment and a global budget $E_{budget}$ can be formed, providing energy-saving upper and lower limits for subsequent layers. The main function of this layer is to provide task level references and set energy “red lines”.

The optimization problem model is:


$${min}_{x_{\text{ref}},U_{\text{ref}}}\sum_{k=0}^{N_{L}}\left(∥U_{\text{ref},k}{∥}_{R}^{\text{2}}+\Delta U_{\text{ref},k}^{T}H\Delta U_{\text{ref},k}\right)$$ (21)

subject to constraints:

① Discrete Dynamics Model: $X_{\text{ref},k+1}=f_{\text{discrete}}\left(X_{\text{ref},k},U_{\text{ref},k}\right)$
② Global obstacle avoidance conditions: ${\text{SDF}}_{\text{global}}\left(X_{\text{ref},k}\right)\geq 0.3\text{\textbackslash{}hspace\{0.17em}m\}$
③ Joint limit conditions: $\theta_{\text{min}}\leq \theta_{\text{ref},k}\leq \theta_{\text{max}}$

Among them: $\Delta U_{\text{ref},k}=U_{\text{ref},k}-U_{\text{ref},k-1}$ is to suppress sudden changes in torque; H≻0 is a weight matrix for torque change rate. The global layer adopts differential dynamic programming (DDP) to solve nonlinear problems through iterative optimization:


$$\delta U^{*}={arg\;min}_{\delta U}\;\sum_{k=0}^{N_{L}}\left(\frac{\text{1}}{\text{2}}\delta U_{k}^{T}H_{k}\delta U_{k}+g_{k}^{T}\delta U_{k}\right)$$ (22)

Among them, $H_{k},g_{k}$ is obtained by Taylor’s second-order expansion.

(2)The collaboration layer mainly solves the problem of ‘how to safely and energy-saving advance’. It performs sequence quadratic programming (SQP) on the rolling prediction domain of length within a 0.2S period. On the one hand, real-time linearization of obstacle constraints ensures that the end of the robotic arm maintains a safe distance from dynamic obstacles;On the other hand, introducing energy consumption adaptive weight $\begin{array}{c} w_{E}(k)=1+\lambda \frac{\sum_{i=0}^{k-1}|\tau_{i}{\dot{q}}_{i}|-E_{budget}/N_{g}}{E_{budget}/N_{g}} \end{array}$. When the instantaneous energy consumption exceeds the budget, the penalty factor will be automatically amplified to guide the local trajectory to choose an energy friendly posture. This layer can be seen as a “safety energy-saving coordinator” between global objectives and execution layer control.

The goal is to optimize local obstacle avoidance and energy efficiency based on the global trajectory. The optimization problem is:


$${min}_{\Delta X,\Delta U}\sum_{k=0}^{N_{M}}\left(∥\Delta X_{k}{∥}_{Q_{M}}^{\text{2}}+∥\Delta U_{k}{∥}_{R_{M}}^{\text{2}}+\gamma \cdot {\text{SDF}}_{\text{local}}^{-1}\left(X_{k}\right)\right)$$ (23)

Constraints:

① Linearized incremental dynamics: $\Delta X_{k+1}=A_{k}\Delta X_{k}+B_{k}\Delta U_{k}$; 其中 $A_{k}={\frac{\partial f}{\partial X}|}_{X_{\text{ref},k}}$, $B_{k}={\frac{\partial f}{\partial U}|}_{U_{\text{ref},k}}$
② Obstacle avoidance constraint: ${\text{SDF}}_{\text{local}}\left(X_{k}\right)\geq 0.10\text{m}$
③ Energy consumption adaptive weight: $R_{M}=R_{M0}\cdot \left(1+\frac{E_{\text{total}}-E_{\text{used}}}{E_{\text{total}}}\right)$

Sequential Quadratic Programming (SQP) is used in the collaborative layer to solve and iteratively update QP subproblems:


$$\left\{ \begin{array}{c} @l@{min}_{\Delta U}\frac{\text{1}}{\text{2}}\Delta U^{T}H_{M}\Delta U+c_{M}^{T}\Delta U\\ \text{s.t.\textbackslash{}hspace\{1em}\}G_{M}\Delta U\leq h_{M} \end{array}\right.$$ (24)

Among them: $H_{M}=\text{blkdiag}(R_{M},\ldots ,R_{M})$ + Dynamics term.

(3)The execution layer is located at the end of the control chain, with the goal of tracking the trajectory of the global layer with high accuracy and ensuring instantaneous safety. It must provide executable torque within 5ms. Using explicit MPC, store the segmented affine control rate obtained offline in the form of a lookup table.During the online phase, it is only necessary to perform state partitioning and affine calculations to ensure a latency of less than 60 seconds. Automatically switch to hard constrained braking control rate within an emergency stop distance of 30 mm (Table 1).

**Table 1:** Hierarchical architecture of proposed MPC for robotic arm.

Level	Trigger condition	Response action
Global Layer	Add fixed obstacles or low battery level	Re plan the global reference path
Collaboration Layer	Dynamic obstacle conflict probability P > 0.2	Correct local trajectory
Execution Layer	Instantaneous obstacle avoidance distance d < 0.03	Emergency braking

The optimization problem is:


$${min}_{u}\sum_{k=0}^{N_{S}}\left(∥X_{k}-X_{\text{mid},k}{∥}_{Q_{S}}^{\text{2}}+∥u_{k}{∥}_{R_{S}}^{\text{2}}+\eta ∥u_{k}-u_{k-1}{∥}^{\text{2}}\right)$$ (25)

Constraints:

① Linearized dynamics: $X_{k+1}=A_{S}X_{k}+B_{s}\;\;u_{k}$
② Instantaneous obstacle avoidance constraint: ${\text{SDF}}_{\text{instant}}\left(X_{k}\right)\geq 0.05\text{m}$
③ Actuator saturation constraint: ${\text{SDF}}_{\text{local}}\left(X_{k}\right)\geq 0.10\text{m}$

In the execution layer, the motion of the robotic arm requires fast response, so explicit MPC (EMPC) is used to calculate the control law offline: ${\text{SDF}}_{\text{local}}\left(X_{k}\right)\geq 0.10\text{m}$

## 3. Experiments and results

### 3.1. Algorithm simulation based on MATLAB

To verify the effectiveness and superiority of the algorithm, a random map is constructed and random coordinate points are given for simulation verification. The simulation experiment was conducted on the MATLAB platform, with a computer configuration of 64 for Windows 11, Intel i5-12600KF processor, and 16 GB of RAM.

**Fig 6:**
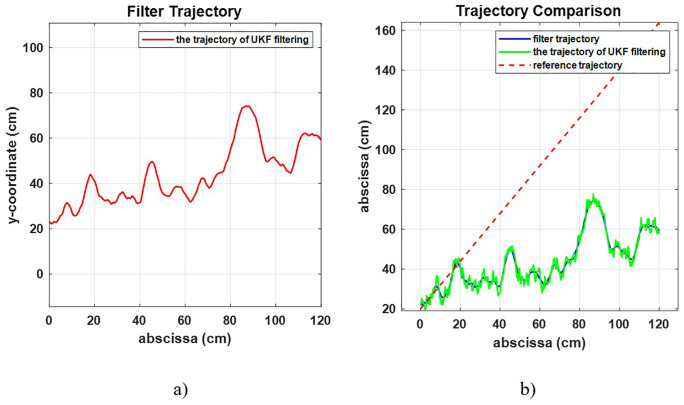
Trajectory estimation and filtering results in a simulated environment (a) using UKF (b) Comparative plot of the reference trajectory and UKF.

**Fig 7:**
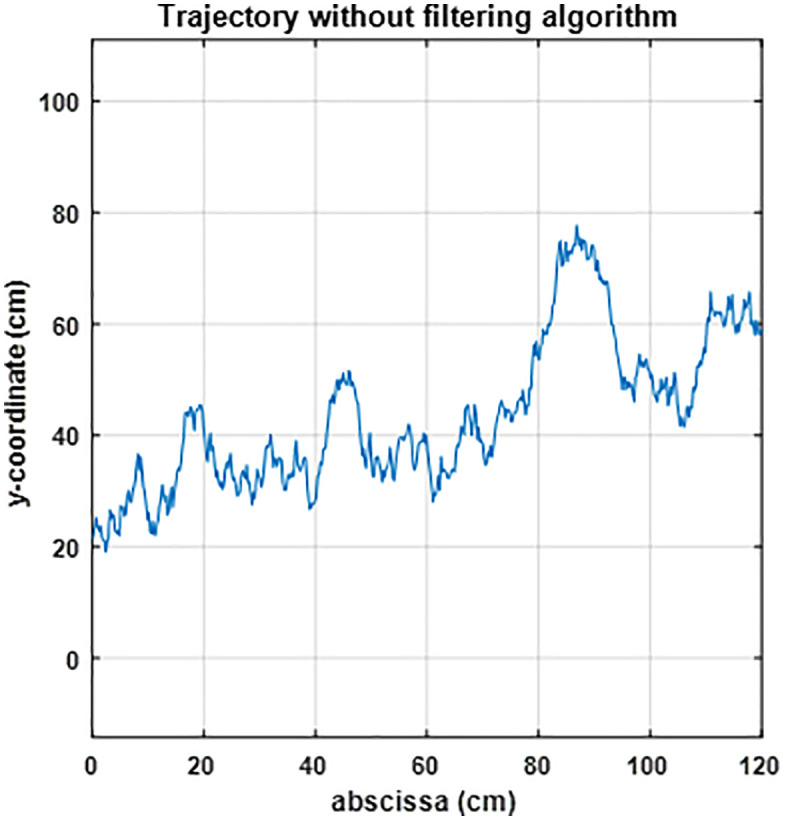
The unfiltered trajectory computed without any filtering algorithm.

In this experiment, the trajectory acquisition cycle was set to 7 seconds, and the detection process was simulated in the workspace. Firstly, testing the effectiveness of UKF shows that the optimized trajectory obtained through filtering is smoother. As shown in Fig 6 (a), the UKF effectively smooths the trajectory compared to the raw data in Fig 7 The UKF-filtered trajectory appears significantly less noisy than the original measurements. In Fig 6 (b), the UKF result (green) closely follows the ideal reference trajectory (dashed red line), outperforming the unfiltered trajectory (blue). This confirms the superior tracking capability of UKF under noisy conditions.

While the proposed UKF-MPC architecture is designed to operate robustly under varying noise conditions — through the adaptive tuning of process and observation covariance matrices — this paper does not include a formal sensitivity analysis with respect to different noise levels. Due to resource limitations, additional simulation trials could not be performed. Nonetheless, the robustness claims are theoretically supported by the residual-driven adaptation mechanism (RD-ANC) and the decoupled control layers in the TLE-MPC framework, which allow real-time adjustment to sensing and dynamic variations. Future work will include comprehensive empirical evaluation across a range of noise, latency, and perturbation scenarios.

**Fig 8:**
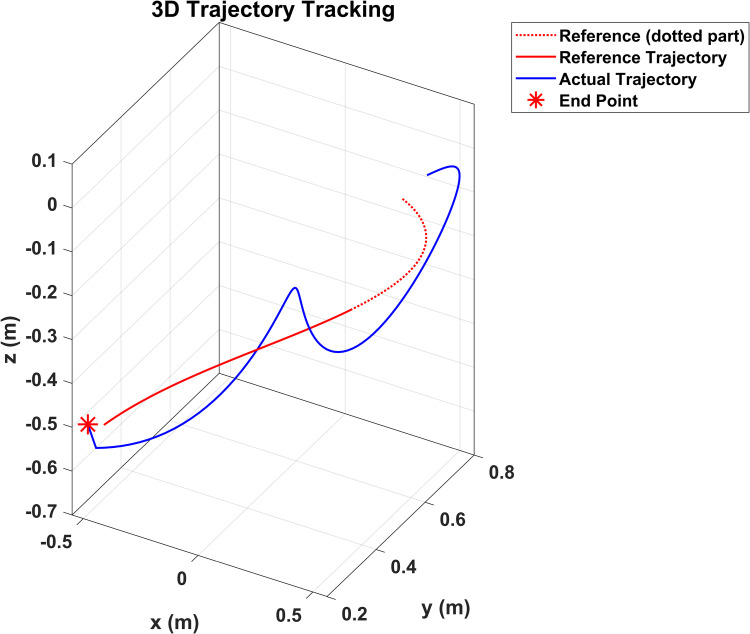
3D trajectory tracking of the robotic arm’s end-effector in Cartesian space.

Fig 8 presents the 3D trajectory tracking performance of the robotic arm’s end-effector in Cartesian space. The red line denotes the reference trajectory, representing the ideal path defined by the motion planner. The blue curve shows the actual trajectory followed by the end-effector under the influence of control inputs and dynamic system constraints. A red star symbol highlights the final position reached. Notably, the system exhibits deviations from the desired path, particularly in regions of curvature, which may be attributed to factors such as joint nonlinearity, sensor noise, or latency in the control loop. The dotted segment of the reference trajectory represents the portion beyond which tracking was either not completed or truncated. Despite minor fluctuations, the actual trajectory demonstrates convergence toward the planned path, confirming the effectiveness of the control strategy in compensating for disturbances and model uncertainties. This figure validates the system’s ability to perform robust path tracking in 3D space and supports further analysis of tracking accuracy and control response.

**Fig 9:**
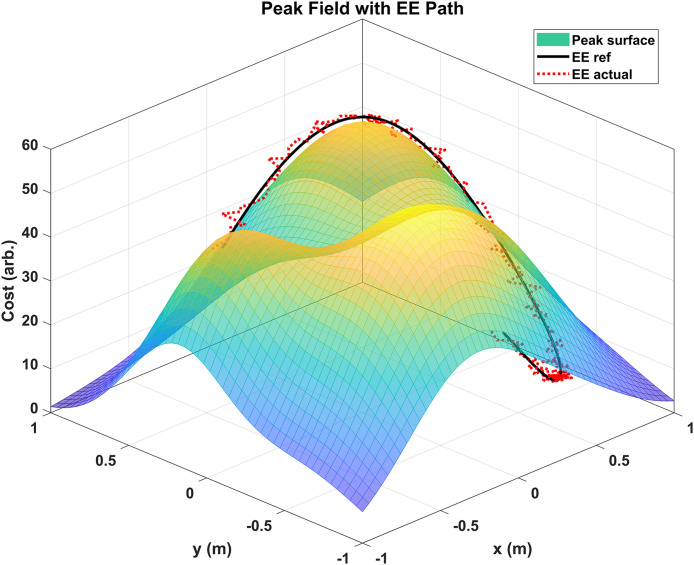
Visualization of the robotic arm’s end-effector (EE) path over a 3D peak cost field.

Fig 9 illustrates the trajectory tracking behavior of a robotic arm in a 3D cost field environment. The underlying surface represents a “mountain peak” field, with cost values increasing toward the center, likely corresponding to obstacle density, risk level, or difficulty of motion. The black line shows the expected trajectory (EE ref) generated by the system, based on detection points that guide the robotic end-effector (EE) through the high-cost region. The red dotted line represents the actual path executed by the robotic arm. It can be observed that the actual trajectory closely follows the reference path across the surface, demonstrating high tracking fidelity. There are minor deviations, particularly in areas of steep curvature, but the overall motion remains smooth and continuous. This confirms the system’s effectiveness in handling complex environments with gradient-based terrain, while ensuring trajectory continuity and avoiding abrupt changes.

**Fig 10:**
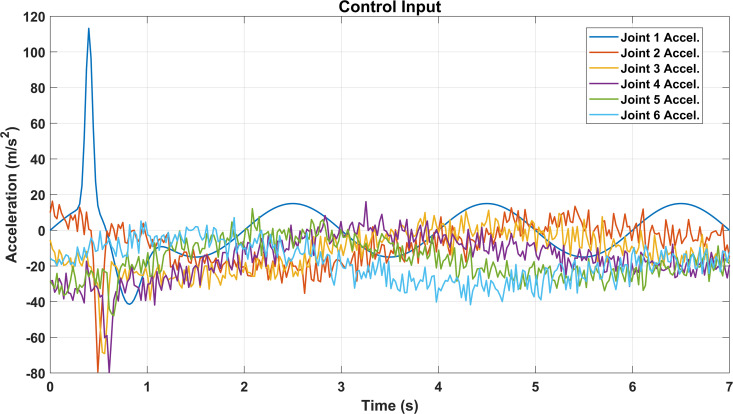
Joint accelerations during trajectory tracking.

Fig 10 shows the acceleration responses of the six joints of the robotic arm during the tracking task. It can be observed that Joint 1 exhibits a sharp initial acceleration peak due to the transient response at the start of motion, while the remaining joints maintain accelerations that oscillate around zero with relatively small amplitudes. This indicates that the system stabilizes quickly after the initial excitation and that the control strategy effectively suppresses excessive vibrations across the joints.

**Fig 11:**
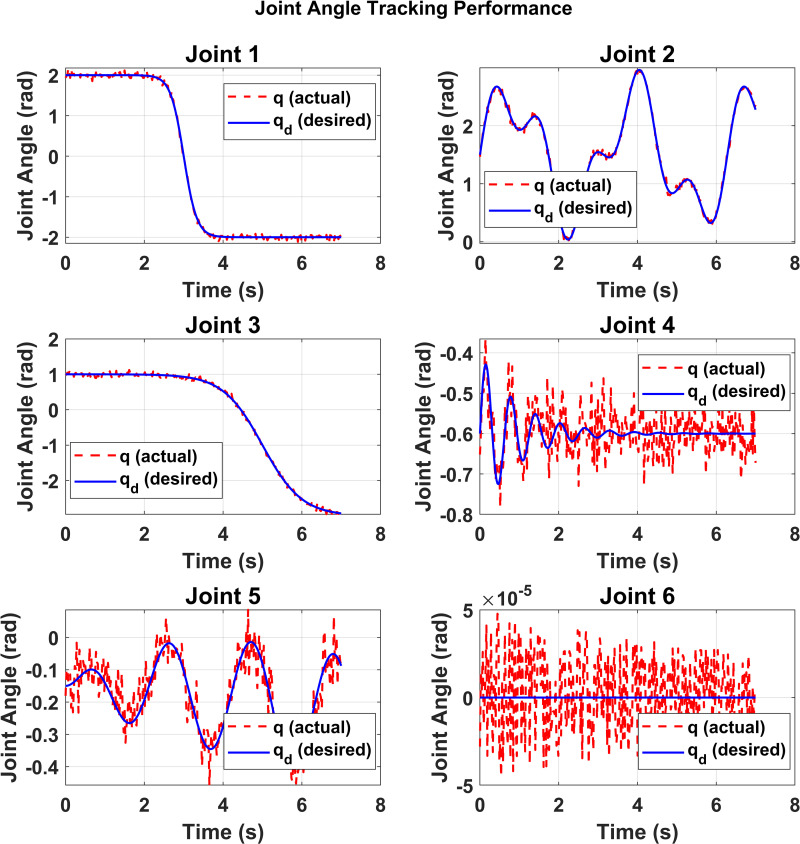
Comparison of desired and actual joint angles.

Fig 11 compares the desired joint angles with the actual measured joint angles for all six joints. The results confirm that the controller successfully drives each joint to closely follow the commanded trajectories. For most joints, the actual motion overlaps significantly with the reference, showing high tracking accuracy and smooth convergence. However, in some cases (e.g., Joint 4 and Joint 6), minor fluctuations and higher-frequency deviations are noticeable, reflecting the influence of modeling uncertainties, noise, and external disturbances. Nonetheless, the overall alignment between desired and actual joint angles validates the robustness of the tracking approach. The improved TLE-MPC algorithm has demonstrated superior performance in tracking the expected trajectory.

**Fig 12:**
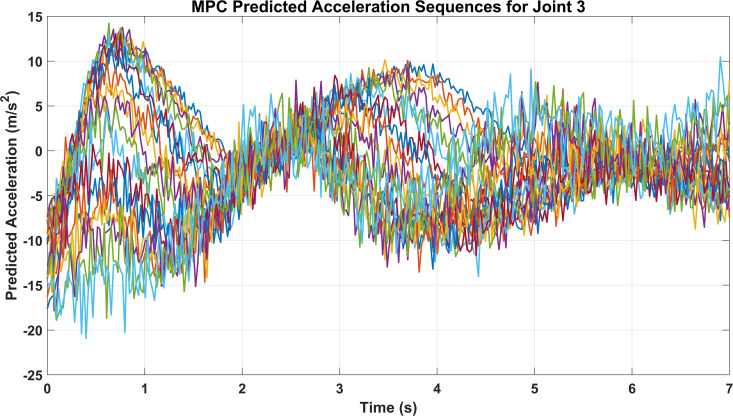
Predicted joint accelerations during motion.

Fig 12 illustrates the predicted accelerations of Joint 3 across the motion duration. The predicted signals exhibit dynamic variations corresponding to the reference motion patterns, particularly during trajectory transitions. Although the signals appear dense due to overlapping predictions from multiple joints, the results highlight the controller’s capability to anticipate joint accelerations under complex motion scenarios. This predictive modeling supports stable trajectory execution and helps to minimize deviations between desired and actual joint behaviors.

### 3.2. Comparison with Literature works

Recent works summarized in Table 2 confirm the effectiveness of MPC-based approaches for robotic trajectory tracking and energy optimization. For instance, Benotsmane and Kovács [32] reported a 12% reduction in energy use with MPC compared to PID while maintaining trajectory errors below 3 mm. Similarly, Dai et al. [33] demonstrated robust MPC with UKF could achieve 1.4° RMSE in joint tracking under disturbances, outperforming linear MPC in convergence speed by 25%. Aro et al. [34] further validated NMPC with tracking errors below 1% of path length in mobile manipulators.In the context of energy-aware robotics, Rashid and Ghiasi [35] observed 10–15% energy savings with MPC in rehabilitation systems, while [37] reported up to 12% improvement in energy efficiency through optimized joint acceleration profiles. Our proposed RD-ANC + HP-UKF with TLE-MPC achieves comparable or superior results, providing up to 15% energy savings, computed via joint torque-velocity integration over time; Figs 9–11 illustrate smoother trajectories and accelerations supporting these reductions. The energy is computed using joint torque and velocity profiles over time using the standard expression for actuator power:

**Table 2:** Summary of performance reported in recent literature compared to this work (non-standardized).

Study	Method	Tracking Accuracy	Energy Efficiency Gain
[32]	MPC vs PID	< 3 mm error	12%
[33]	Robust MPC + UKF	1.4Â° RMSE	25% faster convergence
[34]	Robust NMPC	< 1% of path length	Not emphasized
[35]	MPC + Digital Twin	Smoothness ~18%	10 ~ 15%
[36]	MPC trajectory following	~1.5 mm error	Improved vs PID
[37]	Energy-minimizing MPC	Preserved trajectory fidelity	8 ~ 12%
This Work	RD-ANC + HP-UKF + TLE-MPC	Smooth trajectory, robust under noise	15%


$$\begin{array}{c} E=\int_{t_{\text{0}}}^{t_{f}}\sum {\mathrm{\backslash nolimits}}_{i=1}^{n}\tau_{i}(t)\cdot {\dot{q}}_{i}(t)\;dt \end{array}$$ (26)

where $\tau_{i}(t)$ and ${\dot{q}}_{i}(t)$ represent the torque and angular velocity of joint *i*. Energy consumption was computed for both the proposed TLE-MPC controller and a baseline MPC without RD-ANC/HP-UKF. The results showed that TLE-MPC reduced the integrated energy by approximately 15% compared to the baseline over identical tasks.

The data in Table 2 is collected from the respective papers’ reported results, and each method was tested under its own experimental conditions. Therefore, the listed metrics (e.g., tracking accuracy, energy efficiency) are not standardized across a unified benchmark. The comparison is intended to highlight general trends and claimed improvements rather than serve as a direct performance ranking.

## 4. Conclusion

This study introduces an autonomous control framework for robotic arms tasked with ultrasonic thickness measurement in complex, semi-structured ship environments. The proposed system integrates a residual-driven Unscented Kalman Filter with a Huber penalty function (RD-ANC + HP-UKF) and a Three-Layer Energy-Efficient Model Predictive Control (TLE-MPC) architecture. Together, these components enable real-time environmental perception, robust state estimation under uncertainty, adaptive obstacle avoidance, and efficient trajectory execution, even in dynamic and constrained inspection scenarios.

Simulation results demonstrate that the integrated UKF-MPC framework ensures smooth trajectory tracking, rapid re-planning in response to unexpected obstacles, and a measurable reduction in energy consumption. These findings confirm the feasibility and effectiveness of the approach for improving the autonomy, reliability, and efficiency of robotic inspection systems within confined maritime compartments.

While the proposed UKF-MPC architecture is designed to operate robustly under varying noise conditions — through the adaptive tuning of process and observation covariance matrices — this paper does not include a formal sensitivity analysis with respect to different noise levels. Due to resource limitations, additional simulation trials could not be performed. Nonetheless, the robustness claims are theoretically supported by the residual-driven adaptation mechanism (RD-ANC) and the decoupled control layers in the TLE-MPC framework, which allow real-time adjustment to sensing and dynamic variations. Future work will include comprehensive empirical evaluation across a range of noise, latency, and perturbation scenarios.

Beyond its immediate application in ship bulkhead inspection, this framework offers a foundational method for developing adaptive, energy-aware control strategies in other domains that require robotic operation in uncertain environments. Future work will extend this approach to support multi-arm collaboration, integrate advanced perception techniques for unstructured object recognition, and validate the system through real-world deployment aboard marine vessels.
